# Identifying Human Trafficking Victims: A Potential Role for Forensic Dermatology

**DOI:** 10.7759/cureus.48905

**Published:** 2023-11-16

**Authors:** Philip R Cohen

**Affiliations:** 1 Dermatology, University of California, Davis Medical Center, Sacramento, USA; 2 Dermatology, Touro University California College of Osteopathic Medicine, Vallejo, USA

**Keywords:** victims, tattoo, sex, trafficking, labor, infection, human, forensic, disease, dermatology

## Abstract

Human trafficking is a worldwide problem that predominantly affects women and children. The victims are recruited by coercion, deception, or force and then exploited for commercial sex acts or labor, or both. Human trafficking results in severe suffering of the victims including not only physical injuries but also psychological consequences. Many of the victims of human trafficking encounter the medical system; however, this chance for potential intervention is often not realized by the clinician treating the individual. Many of the manifestations of injuries to human trafficking victims involve the skin, hair, nails, and mucosa. Hence, there is a paramount opportunity for forensic dermatology in the detection and evaluation of suspected victims of human trafficking. Cutaneous manifestations frequently observed in victims of sex trafficking include branding (with tattoos), rashes, bruising, and sequelae of self-injurious behavior; in addition, mucocutaneous stigmata of sexually transmitted diseases may be present. Skin features more commonly associated with victims of labor trafficking include deep and long cuts, skin injuries (such as bruises and tears), and scars from prior burns and knife cuts. The presence of an uncommon infection affecting the skin, such as new world leishmaniasis, that only occurs in a specific and restricted geographic endemic area can also be a subtle clue to human trafficking. Forensic dermatology has the potential to identify victims of human trafficking; when a healthcare worker entertains the possibility of human trafficking, a comprehensive cutaneous examination may provide objective evidence that the individual who is being evaluated and treated may be a human trafficking victim and therefore prompt the clinician to initiate appropriate intervention.

## Editorial

Introduction

Human trafficking is a global problem; not only deception, or coercion, but also force is used to exploit and recruit persons for commercial sex acts and labor. Most victims of human trafficking are not reported; this results from language barriers experienced by the victim and from the victim’s fear of both law enforcement and the trafficker. Importantly, individuals who participate in providing medical care can potentially identify and provide resources for human trafficking victims [1-3].

Analysis of the Counter-Trafficking Data Collaborative data pool in the period from 2010 to 2020 showed human trafficking to be most prevalent in the United States, Russia, and the Philippines; a total of 87,003 victims of human trafficking were identified. Trafficking in human beings predominantly occurred in women (70%) and children. The most frequent age groups included children ranging from nine to 17 years (11.9%), followed by victims between the ages of 30 to 38 years (9.8%); however, for more than half of the victims (53%), the age was unknown [1].

Victims of human trafficking can experience severe physical health risks including not only injuries from violence but also infections (such as those resulting from sexually transmitted diseases). Human trafficking victims also suffer from the consequences of detrimental psychological issues (including depression, low self-esteem, fear, shame or guilt, distrust of medical personnel and law enforcement, and isolation from family, friends, and communities), and substance (such as alcohol and drug) abuse. Trafficking in human beings can involve child soldiering, commercial sexual exploitation and forced labor of children and minors, debt bondage or bonded labor, forced labor (in occupations such as agricultural farmworkers, factory workers, or domestic servants) and slavery of adults, forced marriage, organ trafficking, and sex trafficking [1-3].

Forensic science is used to examine evidence and investigate crimes. The observations may be presented in a court of law. Scientific methods and expertise of individuals trained in various disciplines are used to perform the evaluation.

Forensic dermatology can be used to determine the etiology of an injury. A complete examination of the skin, hair, nails, and mucous membranes may be performed. Skin findings of abuse, neglect, or torture in victims of human trafficking may be discovered, including bruises, physical injuries, and tattoos. In addition, submissive answers, rehearsed responses, and unusual patient behavior by the individual during the clinician-patient encounter may prompt the clinician to consider additional investigation that the patient is a victim of human trafficking [3].

Forensic dermatology may have a crucial role in the detection and evaluation of suspected victims of human trafficking; a comprehensive evaluation during the initial visit is critical since many human trafficking victims do not return to the same clinician for a second visit because of a lack of comfort in the clinician-patient interaction. Tattoos, injuries to the skin, and cutaneous infections may be the presenting features of victims of human trafficking. Awareness of the stigmata of potential manifestations of cutaneous and mucosal injuries observed in victims of human trafficking, especially if they do not correlate with the individual’s health history, may enable clinicians to suspect that the patient is a human trafficking victim. Therefore, to identify human trafficking victims a complete skin examination--including the hair, the nails, and the mucous membranes--is a crucial component of a comprehensive evaluation of the patient [3].

Discussion

Human trafficking victims may be encountered when they seek medical intervention. They may present with injuries or sequelae of physical abuse, psychological abuse, sexual abuse, or substance abuse. The victims most commonly present in emergency departments or urgent care clinics and frequently the presenting problem is managed without recognizing the patient to be a victim of human trafficking [2].

Many of the physical injuries affecting human trafficking victims are cutaneous. Indeed, the findings associated with physical abuse experienced by the victim of human trafficking often affect the skin, hair, nails, and mucosa. Therefore, when human trafficking is suspected, medical personnel should attempt to elicit a comprehensive medical history and perform a thorough examination of the person’s skin and mucous membranes [3].

Many victims of human trafficking are branded (with tattoos) by their traffickers. This is especially prevalent in victims of sex trafficking; in addition, typical findings include not only rashes and bruising but also sequelae of self-injurious behavior may be present. The tattoos may be located on highly visible areas such as the breasts, eyelids, face, or neck. Alternatively, they may be placed in private or hidden areas including the genitalia or oral mucosa [3,4].

The tattoos are a designation of ownership. They can include the names or initials of the traffickers. They can also be distinctive symbols (such as weapons) or depictions of wealth (such as bar codes, currency symbols, or money bags). Observation of unusual tattoos that are found in atypical locations might prompt the consideration that the individual is a victim of human trafficking [3,4].

Sex trafficking survivors are branded by these tattoos. Some of the survivors attempt to cover up their branding tattoos by receiving secondary tattoos. Alternatively, clinicians with expertise using lasers have been able to make a significant positive impact on the recovery of sex trafficking survivors by laser removal of the branding tattoo [4].

Physical trauma, abuse, or torture involving the breasts and genitalia of women may be experienced by victims of sex trafficking. Particularly in victims of labor trafficking, cuts, burns, and skin injury are likely to be found. For example, evidence of either acute trauma such as deep and long cuts and cutaneous injuries (including bruises and tears), or chronic injuries including scars from previous burns or knife cuts may be detected during a complete physical examination; in addition to tattoos (consistent with branding, especially if observed in a minor), other common findings on skin evaluation include rashes, sores, and pruritus. When the reported etiology is not readily compatible with the visualized findings, the possibility of human trafficking should be suspected [3].

The development of sexually transmitted infections in a sex trafficking victim may prompt the trafficker to take the victim for medical treatment; indeed, while being exploited, 50% to 80% of trafficked human beings seek health care. Mucocutaneous features of diseases acquired by sexual contact, when evaluating the groin, should raise suspicion of possible human trafficking; these may include a painless genital ulcer (of primary syphilis), a painful genital ulcer (of chancroid), massively enlarged inguinal lymph nodes (of lymphogranuloma venereum), painful erythematous-based grouped vesicles (of recurrent herpes simplex virus infection), umbilicated papules (of molluscum contagiosum), a filiform papillomatous lesion (of human papillomavirus condyloma acuminatum infection). In addition, secondary syphilis can display various presentations not only as moist verrucous papules (of condyloma lata) or lesions on the palm and soles, but also generalized cutaneous papulosquamous eruptions mimicking either common dermatosis (such as pityriasis rosea) or erythematous nodules (masquerading as cutaneous lymphoma) [2,3].

Forensic dermatology had a seminal role in the discovery of new world cutaneous leishmaniasis and its association with an extensive human trafficking route. The trafficking route involved multiple nations initially by air travel (four flights) originating in Djibouti city (Djibouti), to Dubai (United Arab Emirates), to Moscow (Russia), to Havana (Cuba), to Quito (Ecuador), and then by a ground route through Ecuador and Columbia to the Columbian/Panamanian border and subsequently to Mexico. Four Somali and one Ethiopian had been apprehended and incarcerated in Mexico during the previous two months by the Immigration and Customs Enforcement agents. Each of the five men had the same route of travel and at least one skin ulcer which initially was either a nodule or pustule; they eventually received a medical assessment in San Diego, California (United States) [5].

The men were evaluated by physicians, including dermatology and infectious disease specialists. A punch biopsy of an ulcer on each of the men was performed for microscopic evaluation and cultures. All the biopsies not only demonstrated pathologic findings of cutaneous leishmaniasis but also grew *Leishmania panamensis* [5].

The men were from regions of East Africa endemic for old world *Leishmania *species that could result in both cutaneous and visceral disease. However, the identification of *L. panamensis* established that the men acquired their infection in either Ecuador, Colombia, or Panama after exposure to the vector for this infection by the *Lutzomyia* species of sand fly. Additional history regarding the ground travel across Panama revealed that the men camped outside and experienced multiple bites by insects smaller than mosquitoes [5].

Forensic dermatology was paramount in discovering the ground route of human trafficking that had occurred through a region endemic to *L.* *panamensis*. Initially, the men had not been forthcoming with the details of their trafficking route. However, the dermatologic forensic evaluation provided the evidence of an infection that documented the location of their exposure to the vector for their protozoan infection [5].

Conclusion

Victims of human trafficking are often not recognized to be victims by the treating clinician when they seek treatment. Human sex trafficking victims frequently experience branding (with tattoos), mucocutaneous features of sexually transmitted diseases, rashes, bruising, and sequelae of self-injurious behavior. Deep and long cuts, skin injuries (such as bruises and tears), and scars from prior burns and knife cuts are skin manifestations commonly noted in victims of labor trafficking. In addition, the discovery of a cutaneous infection, that has a limited endemic geographic distribution, can provide essential information that results in the discovery of human trafficking. In conclusion, forensic dermatology can have a critical role in the detection and evaluation of suspected victims of human trafficking; a comprehensive cutaneous examination may demonstrate findings suggestive of human trafficking, thereby prompting the clinician to initiate appropriate intervention.
